# Protection of *Aronia melanocarpa* Fruit Extract from Sodium-Iodate-Induced Damages in Rat Retina

**DOI:** 10.3390/nu13124411

**Published:** 2021-12-09

**Authors:** Yan Xing, Shan Liang, Yuanyuan Zhao, Shuo Yang, He Ni, Haihang Li

**Affiliations:** 1 Guangdong Provincial Key Lab of Biotechnology for Plant Development, School of Life Sciences, South China Normal University, Guangzhou 510631, China; xingyan135@163.com (Y.X.); 20131032@m.scnu.edu.cn (H.N.); 2 Key Laboratory of Microbial Physiological and Metabolic Engineering, Institute of Microbiology, Chinese Academy of Sciences, Beijing 100101, China; liangshan223@hotmail.com; 3 State Key Laboratory of Proteomics, Beijing Proteome Research Center, Institute of Lifeomics, National Center for Protein Sciences (The PHOENIX Center), Beijing 102206, China; yuanyuanzhao0@126.com; 4 Guozhen Health Technology (Beijing) Co., Ltd., Beijing 102206, China; yang8236405@msn.com

**Keywords:** age-related macular degeneration (AMD), anthocyanidin, antioxidant effect, crystallin proteins, secondary degeneration

## Abstract

Age-related macular degeneration (AMD) is one of the major causes of blindness in elderly populations. However, the dry form of AMD has lack of effective treatments. The fruits of *Aronia melanocarpa* are rich in anthocyanins. In this study, the protective effects of aronia fruit extract on rat retina were investigated using a NaIO_3_-induced dry AMD model. Full-field electroretinograms (ERGs) showed that b-wave amplitudes were significantly decreased and the retina structures were disordered in the model. The extract treatment alleviated the injuries. The b-wave amplitudes increased 61.5% in Scotopic 0.01ERG, 122.0% in Photopic 3.0ERG, and 106.8% in Photopic 3.0 flicker; the retina structure disorder was improved with the thickness of outer nuclear layer increasing by 44.1%; and the malonaldehyde level was significantly reduced in extract-treated rat retinas compared to the model. The proteomics analysis showed the expressions of five crystallin proteins, α-crystallin A chain, β-crystallin B2, β-crystallin A3, α-crystallin B chain, and γ-crystallin S, which protect retina ganglion cells, were increased by 7.38-, 7.74-, 15.30-, 4.86-, and 9.14-fold, respectively, in the extract treatment compared to the control, which was also confirmed by immunoblotting. The results suggest that aronia fruit extract, probably due to its anthocyanins, could protect the rat retina by alleviating oxidative damages and by upregulating the crystallin proteins to protect its nerve system.

## 1. Introduction

Age-related macular degeneration (AMD) is known as a progressive blinding disease. There are over 170 million people suffering from the AMD globally [1].

While current treatment is effective for the neovascular or “wet” form of AMD, no therapy is successful for the non-neovascular or “dry” form [2]. Dry AMD accounts for approximately 90% of the total number of people with this vision-threatening condition [3]. It is essential to develop a method to prevent the occurrence and development of dry AMD.

The characters of AMD development include progressive macular degeneration caused by oxidative damage of the retinal pigment epithelium (RPE), which is associated with the degeneration of photoreceptors [4,5,6]. Research on the mechanisms of AMD suggests that the major damages of the disease are due to oxidative stress/damages to the RPE [4,5,6]. Therefore, some studies on the treatments of AMD focus on the suppression of oxidative stress using high doses of antioxidant vitamins and zinc supplements [7,8]. There are studies suggesting that AMD could also lead to damages in the nerve system of retina. A clinical research result showed a 47% loss of ganglion cell layer (GCL) neurons at the end-stage cases of exudative age-related macular degeneration (EXAMD) [9]. Retinal ganglion cell (RGC) death has also been observed in a rodent model after an almost complete loss of photoreceptors [10].

The RGCs are the afferent neurons of the retina, and through their axons in the optic nerve the visual information is sent to the retinorecipient nuclei in the brain for further analysis. Many researchers have shown that partial optic nerve transection (ponT) to the retina of rats can lead to the over production of reactive oxygen species (ROS) and changes in mitochondrial morphology and function [11,12]. However, at a longer term after ponT, a secondary RGC degeneration is observed, including swelling in myelinated axons and myelin sheath thickening, which could then lead to visual dysfunction [12,13]. Previous studies have shown that alpha and beta crystallins could enhance the survival of RGCs after optic nerve injury [14,15]. A recent study using 2D fluorescence difference gel electrophoresis (DIGE) followed by mass spectrum (MS) on the protein expression in RGCs after partial ponT found that in the RGCs of the non-injury region, the expression of six crystallin proteins were dramatically upregulated compared with the injured region after eight weeks [16]. This suggests that these crystallin proteins may protect the RGCs from secondary degeneration. 

One study showed that bilberry anthocyanins have a protective effect against light- induced oxidative damages in rabbit retina [17]. The anthocyanidin has been reported to have antioxidant activity and could scavenge radicals [18] and has a protective effect on the retina against damages caused by AMD [19]. Studies on anthocyanidin have shown that it could prevent the damage of RPE from oxidative stress by decreasing intracellular ROS [20]. However, most of these studies are focused on cells rather than animals.

The fruits of aronia (*Aronia melanocarpa*) contain high levels of anthocyanidin, which is considered to be the major “effective constituent” for anti-oxidative activity [21]. The rat model is classical for the study of AMD induced either by sodium iodate (NaIO_3_) or intense light [17,22]. In this study, sodium iodate (NaIO_3_), which is widely used to simulate the damages during progressive AMD [23,24,25], was used to treat the rats as an AMD model to create oxidative stress in the rodent model to induce RPE and photoreceptor oxidative damage/cell death. We investigated the protective effect of aronia fruit extract on NaIO_3_-treated rat retina and analyzed its function on preventing the RGCs from secondary degeneration by measuring the expression of the above-mentioned crystallin proteins in rat retina. 

## 2. Materials and Methods

### 2.1. Animals

All animals used for experiments in this study were prepared under the procedures according to the Association for Research in Vision and Ophthalmology (ARVO) Statement for the use of Animals in Ophthalmic and Vision Research. Protocols used in this study have been reviewed and approved by the Animal Ethics Committee of the Institute of Medicinal Plant Development (No. SLXD-20201218031). Male Sprague-Dawley (SD) rats 180–200 g were provided by the National Institutes for Food and Drug Control (Beijing, China). The animals were kept at 22 °C and in 12 h/12 h of light/dark cycle condition for a week before being used for experiments.

### 2.2. Aronia Fruit Extract

The Aronia fruit extract used in this study was the purplish red powder purchased from the Greater Hinggan Gebei Frigid Zone Biotechnology Co., Ltd. (Heilongjiang, China). The powder contains 10% starch (exogenously added during preparation of the powder), 10.3% anthocyanidin, and other water-soluble nutrients including saccharides, proteins, and dietary fiber from the fruits of *Aronia melanocarpa*.

### 2.3. Evaluation of Protective Effect of Anthocyanidin against NaIO_3_-Induced Retinal Damage in Rats

A total of 36 SD rats were randomly separated into 3 groups (NOR, MOD, and AC groups), 12 rats each group. In AC group, aronia fruit extract in distilled water was administrated orally once a day using a stomach tube at an equivalent anthocyanidin concentration of 60 mg/kg body weight (the amount of Aronia fruit extract powder is 600 mg/kg body weight) for 28 days. A single treatment of NaIO_3_ at 30 mg/kg body weight was intravenous injected to the rats of this group on day 8 after the start of feeding aronia water extract.

**Figure 1:**
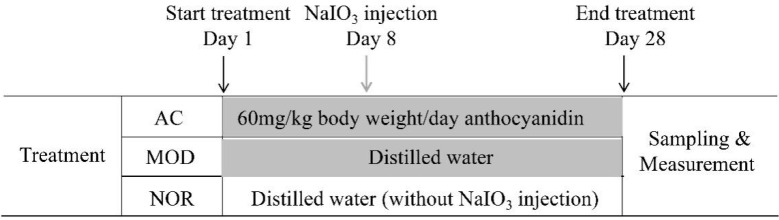
Schematic diagram for the treatment of rats with NaIO_3_ and aronia fruit extract. Thirty-six SD rats were randomly separated into three groups (NOR, MOD, and AC), twelve rats each group. NOR: control without treatment; MOD: damage model by 30 mg/kg body weight NaIO_3_ tail vein injection; AC: anthocyanidin (60 mg/kg body weight) treatment of the damage model.

Distilled water at equivalent amount of aronia fruit extract solution was given orally to the rats of the other two groups. In MOD group, rats were injected with NaIO_3_ at 30 mg/kg body weight on day 8. This established the dry AMD model. In the NOR group, rats were orally administrated with distilled water without other treatment. On day 29, all rats were collected for experiments (Figure 1). 

### 2.4. Electroretinograms

The full-field electroretinograms (ERGs) of rats from each group were recorded using an ERG recording system (D430 Diagnosis, USA). Before the measurement of ERG, the rats were dark-adapted for 24 h [26]. A mixture of ketamine hydrochloride and xylazine hydrochloride at the dosages of 100 mg/kg and 15 mg/kg, respectively, was intramuscularly injected to induce anesthesia of the rats. Eye drops containing 0.5% tropicamide and 0.5% phenylephrine hydrochloride were administered to the eyes of rats for 20 min to dilate the pupils. Light-emitting diodes (LED) electrodes were placed on both corneas. An identical reference electrode was placed under the middle scalp, and a ground electrode was placed subcutaneous of both lower limbs of tested rats. ERGs of Scotopic 0.01ERG, Scotopic 3.0ERG, Scotopic 3.0 oscillatory potentials, Photopic 3.0ERG, and Photopic 3.0 flicker were conducted. Both peak times and amplitudes of a- and b-waves were recorded, and the b-wave amplitudes were statistically analyzed. Twelve samples from each group of rats were measured.

### 2.5. Hematoxylin and Eosin (H&E) Staining of the Retina

Both eyes of rats in each group were removed after euthanasia. The eyeballs were enucleated and fixed in 4% paraformaldehyde containing 20% isopropanol, 2% trichloroacetic acid, and 2% zinc chloride at room temperature for 2 h. After removing the cornea, iris, crystalline lens, and partial vitreous body, the remaining part of the eyes were dehydrated with alcohol for 24 h and then embedded in paraffin. The whole retina samples were cut into 4-µm-thick sagittal sections, and stained with H&E. For each section, digitized images of the entire retina were taken with a digital camera (Leica DMi8, Wetzlar, Germany) at 200× magnification. The thickness of the outer nuclear layer (ONL) was measured with Image J software (US National Institutes of Health, Bethesda, MD, USA). Twelve locations for each retinal section were measured, starting from either side of the optic nerve, with each segment 0.5 mm apart. The 12 measurements were averaged for the mean ONL thickness [27]. 

### 2.6. Measuring Malondialdehyde (MDA)

Venous blood was collected from rats of each group and centrifuged with a bench top centrifuge (Eppendorf, Hamburg, Germany) at 3000 rpm for 10 min, and the supernatant of each sample was used for MDA concentration measurements. The retina samples taken from the eyeballs of each group of rats were homogenated with phosphate buffer saline (PBS, pH 7.4) and centrifuged at 3500 rpm/min for 10 min, and the supernatant was collected for MDA measurement. The MDA level in the supernatant of each sample was determined spectrophotometrically at the wavelength of 532 nm using the measuring kit (Nanjing Jiancheng Bioengineering Institute, Nanjing, China, #20210304). Eight samples were detected from each group of rats. 

### 2.7. Mass Spectrometry Analysis of Retina Proteins

Rat retina proteins were identified and analyzed using tandem MS following the previous protocol [28]. Three retina samples from each group of rats were prepared. The retina tissues from each group were lysed by sonication in 8M urea buffer. After digested with trypsin, the peptides were analyzed on an Orbitrap Q Ex-active HF mass spectrometer coupled with an online EASY-nLC 1200 nano-high-performance liquid chromatography (HPLC) system (Thermo Fisher Scientific, Waltham, MA, USA). The peptide mixtures were separated on a reversed-phase nano-HPLC C18 column (precolumn: 0.1 × 20 mm, 3 μm; analytical column: 0.15 × 120 mm, 1.9 μm) at a flow rate of 600 nL/min with a 78-min gradient: 6 to 9% solvent B for 2 min, 9 to 13% for 8 min, 13 to 26% for 40 min, 26 to 38% for 20 min, 38 to 100% for 1 min, and 100% for 7 min (solvent A, water; solvent B, acetonitrile; 0.1% formic acid). The electrospray voltage was 2.2 kV. Peptides were analyzed by data-dependent MS/MS acquisition mode with a resolution of 120,000 at full-scan mode and 15,000 at MS/MS mode. The full scan was processed in the Orbitrap from mass/charge ratio 250 to 1800; the top 20 most intense ions in each scan were automatically selected for higher-energy collisional dissociation (HCD) fragmentation with normalized collision energy of 32% and measured in Orbitrap. Typical mass spectrometric conditions were as follows: Automatic gain control targets were 3 × 10^6^ ions for full scans and 2 × 10^5^ for MS/MS scans; the maximum injection time was 35 ms for full scans and 80 ms for MS/MS scans; and dynamic exclusion was used for 18 s. Each sample was analyzed with one technical replicate because of the limited sample volume. Target protein expression in each group of rats was determined by average the measurements of 3 retina samples from each group. The mass spectrometry results were analyzed and quantified using PEAKS Studio (Waterloo, Canada). 

### 2.8. Immunoblotting

The proteins of retina tissues were extracted with NETN buffer (50 mM Tris-HCl, pH 8.0, 150 mM NaCl, 0.2% Nonidet P-40, and 2 mM EDTA), and 20 µg proteins of each sample were separated on 12% SDS-PAGE. After electrophoresis, the proteins were transferred onto polyvinylidene fluoride membranes. The membrane was washed with 5% skimmed milk in Tween/Tris-buffered saline (TBST) to block nonspecific binding and then incubated with primary antibodies against α-crystallin A chain and γ-crystallin S. Immunoblots were performed using SuperSignal Western Pick Plus (#34577, Thermo Scientific) [29].

### 2.9. Statistical Analysis

Results are presented as the mean ± standard deviation. Differences between groups were assessed by one-way ANOVA, followed by Tukey’s test or Kruskal–Wallis nonparametric test, and then by Dunn’s test. *p* < 0.05 was considered statistically significant. All statistical analyses were performed using Prism 8.0 (GraphPad Software, San Diego, CA, USA).

## 3. Results

### 3.1. Aronia Fruit Extract Protection of Rat Retina from the Damage Caused by NaIO_3_

Full-field electroretinography is an objective measure of overall retinal function. ERG is an electrical response of the retina to photic stimulation. A flash of light or bright appearance elicits a biphasic negative/positive waveform. The a-wave originating in the receptor level of rods and cones is the initial large negative wave. The b-wave originating in the mid-retina is the following large positive component. Systemic metabolic disorders usually reduce ERG b-wave amplitudes, particularly the scotopic dim flash ERGs [30,31]. 

**Figure 2:**
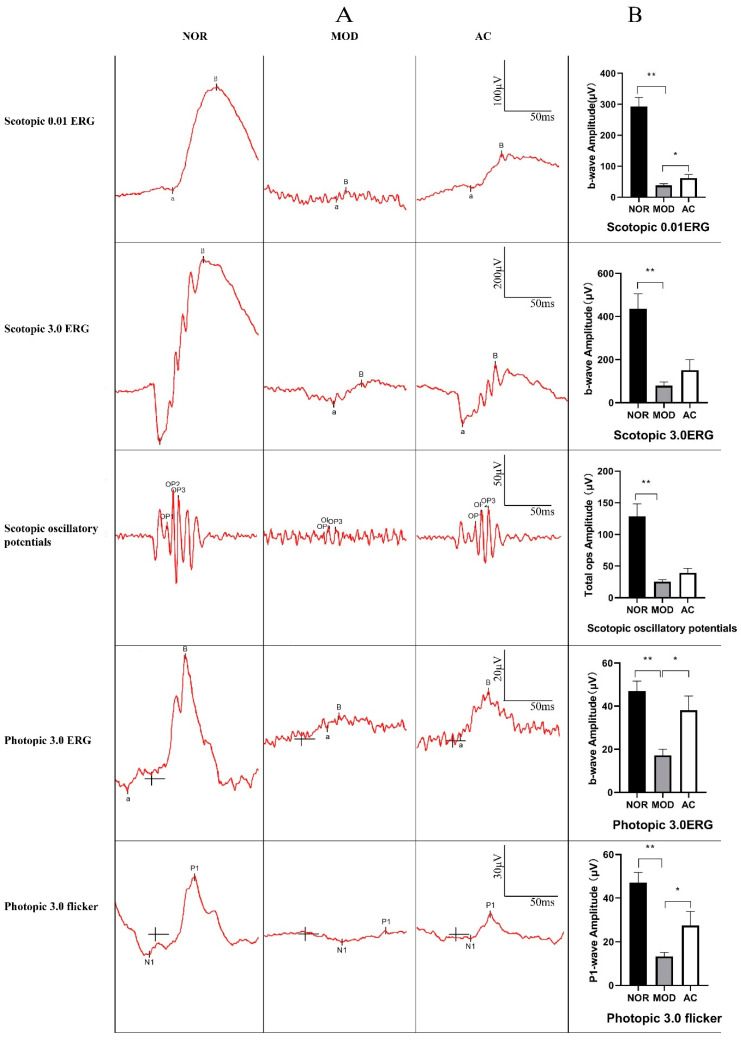
Full-field electroretinograms of the rats by different treatments. Rats from each group were anesthetized and analyzed by electroretinograms (ERGs). (**A**) Full-field ERGs were recorded on the 21th day after NaIO_3_ injection. (**B**) The averages of the three groups of b-wave amplitudes of Scotopic 0.01ERG, Scotopic 3.0ERG, and Photopic 3.0ERG and total amplitude of Scotopic 3.0 oscillatory (3 ops) and P1-wave amplitude. Data of b-wave amplitudes shown are the mean ± standard deviation (*n* = 12). (*) *p* < 0.05, (**) *p* < 0.01 (one-way ANOVA followed by Tukey’s test).

Five different electrical currents in the rat retina of each group were measured according to the ISCEV (International Society of Clinical Electrophysilological Vision) standard. The results showed the ERG b-wave amplitudes for all five different measurements were significantly decreased in the MOD group (*p* < 0.01, Figure 2), compared with those in the NOR group. This indicated a serious damage on the rat retina of the MOD group. The decrease of ERG b-wave amplitudes in Scotopic 0.01ERG, Photopic 3.0ERG, and Photopic 3.0 flicker of the AC group was reduced compared to the MOD group (*p* < 0.05, Figure 2B). According to the ISCEV Standard, Scotopic 0.01ERG indicates a rod-driven response of bipolar cells, Photopic 3.0ERG indicates responses of the cone system, b-wave comes from On- and Off-cone bipolar cells, and Photopic 3.0 flicker is a sensitive cone-pathway-driven response. The results suggested that the damage of retina induced by NaIO_3_ was alleviated in the AC group, and aronia fruit extract showed a potential protective effect on rat retina, especially on cone system.

**Figure 3:**
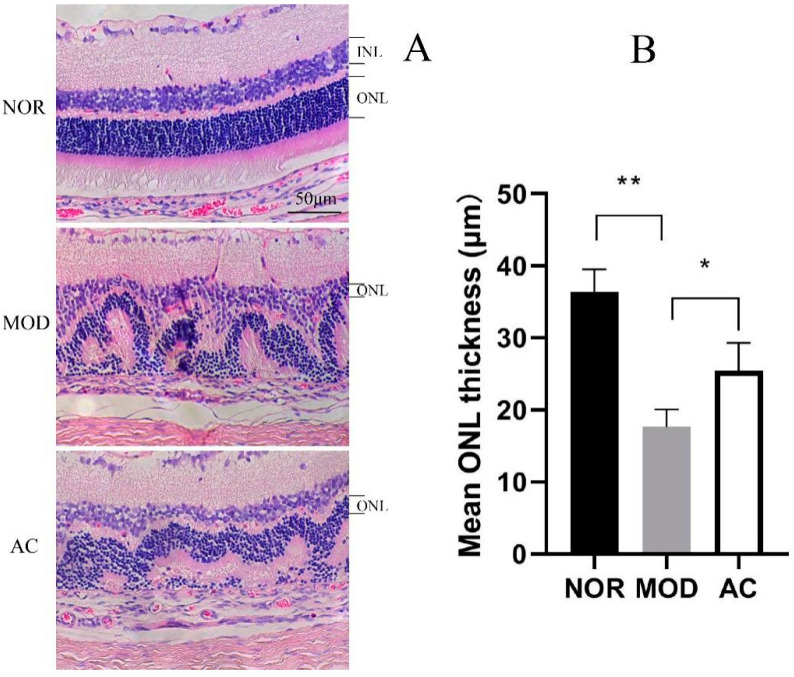
Effect of aronia fruit extract on the structure and outer nuclear layer (ONL) thickness of rat retina. (**A**) Images of the H&E stained sections of rat retinas, taken at 200× magnification. (**B**) Outer nuclear layer thickness of the retinas. Data are the mean ± standard deviations (*n* = 8). (*) *p* < 0.05, (**) *p* < 0.01 (one-way ANOVA followed by Tukey’s test).

The pictures stained with the H&E of rat retina show that the NOR group has a clear retina structure and layers, and the cell nuclei were neatly aligned. In the MOD sample, the retina showed a disordered structure, and a reduction of cell layers with a messed outer and inner nuclear layer (ONL and INL). In comparison, the AC group showed an improvement on its retina structure and cell layers, the alignment of their nucleus was relatively in order compared with the MOD group (Figure 3A). The NaIO_3_ treatment dramatically reduced the thickness of ONL, suggested a progressive ONL thinning. However, the administration of aronia fruit extract can reduce the damage by NaIO_3_ and maintain the ONL thickness (Figure 3B).

### 3.2. Antioxidative Effect of Aronia Fruit Extract Treatment on the Rat Retinas and Serum

**Figure 4:**
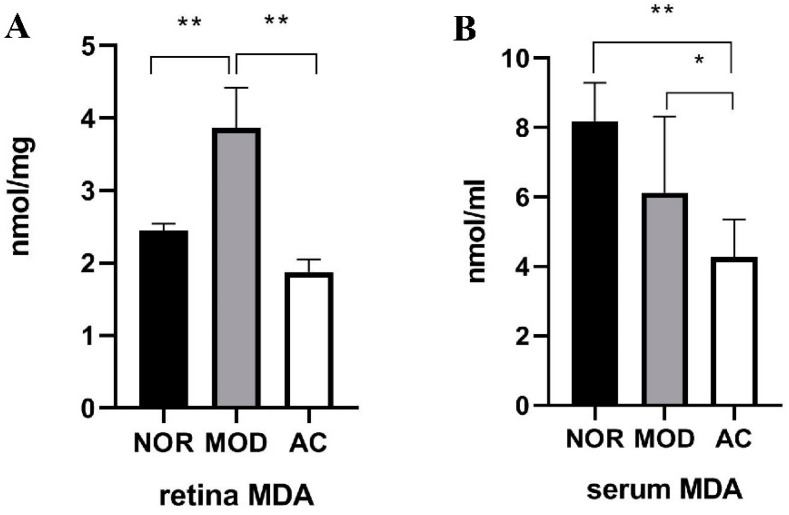
Malondialdehyde (MDA) levels in the retina (**A**) and serum (**B**) of rats. Data are the mean ± standard deviations (*n* = 8). (*) *p* < 0.05, (**) *p* < 0.01 (one-way ANOVA followed by Tukey’s test).

Malonaldehyde (MDA) is a peroxidative product of polyunsaturated fatty acids, and has been widely used as a “biological marker” for measuring oxidative stress [32]. As shown in Figure 4A, the MDA level in the rat retina of NOR group was 2.45 ± 0.19 nmol/mg. NaIO_3_ treatment significantly increased the MDA level to 3.87 ± 0.74 nmol/mg, while the aronia fruit extract treatment significantly reduced the MDA level to 1.88 ± 0.12 nmol/mg in the rat retina (*p* < 0.01, Figure 4A).

The MDA concentrations in the rat serum of the NOR, MOD, and AC groups were 8.18 ± 1.12, 7.31 ± 1.53, and 4.27 ± 1.09 nmol/mL, respectively (Figure 4B). There is no statistical difference between the MOD and the NOR groups, while the serum MDA level of AC group is significantly lower than that of the MOD and NOR groups (*p* < 0.05, Figure 4B). These results suggested that NaIO_3_ treatment caused more oxidative stress in the rat retina but not in rat serum, and the administration of aronia fruit extract could reduce the oxidative stress in both the retina and blood of rats.

### 3.3. Effect of Aronia Fruit Extract Treatment on the Expression of Crystallin Proteins in Rat Retina

**Figure 5:**
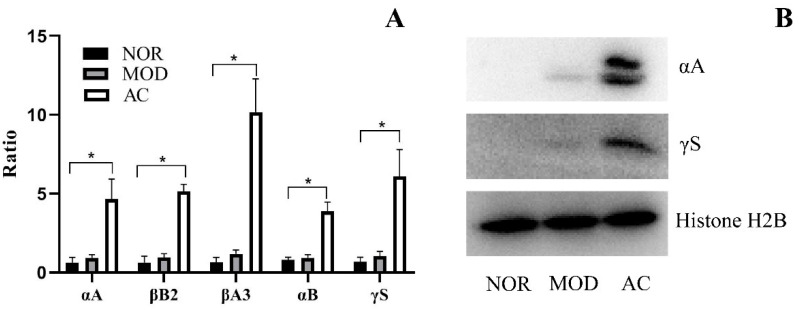
Expression of crystallin proteins in the rat retina. (**A**) Expression of α-crystallin A chain (αA), β-crystallin B2 (βB2), β-crystallin A3 (βA3), α-crystallin B chain (αB), and γ-crystallin S (γS) determined by mass spectrometry. The amount of each protein expressed in No.1 sample in NOR group (NOR-1) was set to be onefold, and the amount of crystallin proteins expressed in other samples were expressed as the ratio of NOR-1. Data shown are the mean ± standard deviations (*n* = 3). (*) *p* < 0.05 (Kruskal–Wallis test followed by Dunn’s test). (**B**) Immunoblotting results showing the presence of α-crystallin A chain and γ-crystallin S in retina sample. The protein histone H2B in each sample was measured as an internal reference to show a basic protein expression in each sample.

A group of six crystallin proteins has been suggested to have certain effects on preventing the secondary degeneration of RGCs in the retina of rats [16]. To investigate the potential effects of aronia fruit extract on rat RGCs, the total protein expressed in the retina samples were analyzed. The results showed that five proteins of the above mentioned crystallin proteins were slightly increased in the rat retina of the MOD group. The expression of α-crystallin A chain (αA), β-crystallin B2 (βB2), β-crystallin A3 (βA3), α-crystallin B chain (αB), and γ-crystallin S (γS) increased 1.48-, 1.43-, 1.84-, 1.17-, and 1.56-fold, respectively, in the MOD group compared with the NOR group (*p* > 0.05, Figure 5A). However, in the retina samples of the AC group, a much higher expression of these crystallin proteins were observed. In the AC group, the expression of five crystallin proteins, αA, βB2, βA3, αB, and γS, were increased by 7.38-, 7.74-, 15.30-, 4.86-, and 9.14-fold, respectively, compared to the NOR group (*p* < 0.05, Figure 5A). The results suggest that aronia fruit extract treatment led to a much higher upregulation of the protective crystallin proteins in the stressed condition.

The amounts of αA and γS in rat retina were detected using immunoblotting. As shown in Figure 5B, both αA and γS were not detected in the NOR group. Very low amounts of the two proteins were detected in the MOD group. However, αA and γS had a significantly higher expression in the rat retina of the AC group, compared to the MOD group. The results confirmed the mass spectrum results shown in Figure 5A.

## 4. Discussion

The fruit of *Aronia melanocarpa* is one of the most abundant sources of anthocyanin, the content of which is up to 460 mg/100 g fresh aronia fruits [33]. In a visible-light-induced retinal degeneration models in pigmented rabbits, the oral administration of bilberry anthocyanins (BAE) had been reported to alleviate the decreasing of ERG b-wave and suppressed the thinning of ONL, and the protective effect was associated with the antioxidative activity [17]. In our results, the alleviation of ERG b-wave decreased together with the suppression of cell layers disorder and ONL thinning were observed in the rats of the AC group treated with aronia fruit extract in comparison with the MOD group (Figure 2 and Figure 3). Anthocyanidin is a group of polyphenolic compounds, and its anti-oxidative activity associated with therapeutic effects in human health have been widely proved [18,20]. The protective effects of aronia fruit extract on the rat retina observed in our ERGs and H&E staining experiments are very likely due to the anti-oxidation activity of anthocyanins. However, we noticed that the protective effects of aronia fruit extract treatment in our study were not as significant as in the previous reports, in which they used 30 to 300 mg/kg body weight of anthocyanidin, proanthocyanidin, or polyphenols in murine retinal degeneration models. The protective effect of anthocyanindin on the retina is dosage-dependent, and its optimal dosage may be about 100 mg/kg [17,34,35,36]. Increasing the dosage of anthocynidin may have better results.

The oxidative damage of the RPE associated with the degeneration of photoreceptors is usually considered to be the characters of AMD development [4,5,6]. Some researchers showed that the nerve system in rat retina could be damaged after the complete loss of photoreceptors [10]. Since the degeneration of photoreceptors is usually considered as a common feature associated with AMD [4,5,6], the RGC death may be suggested as a “secondary” damage of AMD. In the recent study, ponT to the retina of rats has discovered that the upregulation of a group of crystallin proteins including the αA, αB, βA2, βA3, β B2, and γS has a more protective effect on RGCs against secondary degeneration rather than primary degeneration. It was reported that the expressions of the αB and βA2 crystallins were only increased 1.4- and 1.2-fold in the injured region, respectively, while they were increased 3.5- and 2.1-fold of these proteins in the non-injured region [16]. A more significant upregulation of crystallin proteins has been observed in our experiment. In the AC group treated with aronia fruit extract, five crystallin proteins including the αA, βB2, βA3, αB, and γS were up-regulated 7.38-, 7.74-, 15.30-, 4.86-, and 9.14-fold, respectively (Figure 5A), which suggested that the administration of aronia fruit extract may protect the nerve system of rat retinas from the secondary degeneration. Another observation in our experiments is that the expression of these five crystallin proteins were also slightly upregulated in the NaIO_3_-treated MOD group, although there was no statistical difference between the MOD and NOR groups (Figure 5A). These observations were also consistent with our immunoblotting results, which measured the amount of αA and γS in retina samples from each group of rats (Figure 5B). This may suggest that the expression of crystallin proteins is likely to be an innate protective mechanism which protects rat retina nerve cells from secondary degeneration.

The detailed mechanisms for the upregulation of crystallin proteins involving in the suppression of retina nerve system from secondary degeneration remains unknown. Since the crystallin proteins are known as the members of the chaperon protein family, they are usually considered as a part of the protein quality control system in cells and ensuring that “proteins are correctly folded and functional at the right place and time” [37,38,39]. Therefore, one potential protective mechanism of upregulated crystallin proteins and suppressing retina nerve cells degeneration is by regulating protein folding in rat retina. Our mass spectrometry results also showed a downregulation of caspase-3 protein, a factor regulating the apoptosis of RGCs [40], in the retina of the AC group compared with the MOD group (data not shown). The decreased expression of caspase-3 suggests that aronia fruit extract may also protect retina nerve cells from programmed cell death.

## 5. Conclusions

In conclusion, in this study, we successfully established the dry AMD model with NaIO_3_ and found that aronia fruits extract can protect the retina by anti-oxidative activities and upregulating the crystallin proteins expression. Therefore, we could anticipate that supplementing with aronia fruit extract with high anthocyanidin may be an approach to prevent AMD and other damages related to retinal degeneration.
